# Supplementary material for: High-Throughput Drug Screening Revealed That Ciclopirox Olamine Can Engender Gastric Cancer Stem-like Cells
Source: Cancers (Basel). 2023 Sep 3;15(17):4406. doi: 10.3390/cancers15174406 (PMC10487151; doi:10.3390/cancers15174406)
Supplement: Supplementary file 1 [file cancers-15-04406-s001.zip › cancers-2550418-supplementary.pdf]

cancers MDPI

Supplementary Materials

High-Throughput Drug Screening Revealed That Ciclopirox Olamine Can Engender Gastric Cancer Stem-like Cells

Table S1: Primers used for real-time PCR analysis

Gene	Primer Forward (5′ → 3′)	Primer Reverse (5′ → 3′)
**ARNT2**	AAGAACCGGGAGTGGATGTTG	CTGTTGCTGAAGTTGCTTGACG
**β-catenin**	CAATGGCTTGGAATGAGACTGC	GGATCATCCTGGTGATATCCAAG
**c-Jun**	AGTGAGTGACCGCGACTTTT	GATGCCTCCCGCACTCTTAC
**C-MYC**	GCGACTCTGAGGAGGAACAAG	TGGGCTGTGAGGAGGTTTG
**CNOT3**	GGCTCACGAATACCATCGACA	GCTTATCCTTGTCGCCCTTCT
**FOXA1**	AAGGGCATGAAACCAGCGAC	GCCTGAGTTCATGTTGCTGAC
**FOXA2**	ATGCACTCGGCTTCCAGTAT	GTTGCTCACGGAGGAGTAGC
**FOXC2**	CTACAGCTACATCGCGCTCATCA	ACTGGTAGATGCCGTTCAAGGTG
**GATA6**	AAGCGCGTGCCTTCATCA	CATAGCAAGTGGTCTGGGCA
**GLI1**	GAAGTCATACTCACGCCTCGAA	CAGCCAGGGAGCTTACATACAT
**GLI2**	AGCAGCAGCAACTGTCTGAGTGA	GACCTTGCTGCGCTTGTGAA
**Hes1**	ACACGACACCGGATAAACCA	ATGCCGCGAGCTATCTTTCT
**HIF-1α**	GGCGCGAACGACAAGAAAAAGATA	GGCCTTATCAAGATGCGAACTCAC
**HMGA1**	GTGCCAACACCTAAGAGACCT	TCTGCTGGTTTTCCGGCTC
**HMGA2**	CCCAAAGGCAGCAAAAACAA	GCCTCTTGGCCGTTTTTCTC
**HOXA10**	CCCTTCCGAGAGCAGCAAA	TCTTCCGACCACTCTTTGCC
**HOXA5**	GCGCAAGCTGCACATAAGTC	CGGAGAGGCAAAGAGCATGT
**ISL1**	CTGCTTTTCAGCAACTGGTCA	TAGGACTGGCTACCATGCTGT
**KLF4**	CAGAGGAGCCCAAGCCAAAG	TTTCTCACCTGTGTGGGTTCG
**KLF5**	AAGGAGTAACCCCGATTTGG	CAGCCTTCCCAGGTACACTT
**MEIS2**	TCCAGCATCTCACACATCCG	GAAAACCTGCTCGATTTGACTGG
**NANOG**	GAACCTCAGCTACAAACAGGTGAA	TTCTGCGTCACACCATTGCT
**OCT1**	CAAAATGGCGGACGGAGGA	GTTCATTCTTGAGTCTGCTGCTG
**OCT4**	TGCAGCAGATCAGCCACAT	ACACTGGTCCCCCTGAGAAA
**RelA**	GCTGCATCCACAGTTTCCAGA	CCCCACGCTGCTCTTCTAT
**SOX4**	GACTTCGAGTTTGCTCCCCT	TAACTCGCCTTCTTGCTGGG
**SOX9**	CGGAGGAAGTCGGTGAAG	CTGGGATTGCCCCGAGTGCT

**STAT3**	GAGGACTGAGCATCGAGCA	CATGTGATCTGACACCCTGAA
**TCF4**	TTTGGAAGAAGCGGCCAAGAGG	TTGGGGAGGTAGGGGCTCGT
**TEAD2**	TTTGGGGTGTGCCCAGATG	TCCTCACTGCCTTCCTCACT
**ZNF273**	AGCCTAGAAATGGGACCACTG	GCTGTGAAGTGTCCAGGCAT
**18S**	CGCCGCTAGAGGTGAAATTC	CATTCTTGGCAAATGCTTTCG

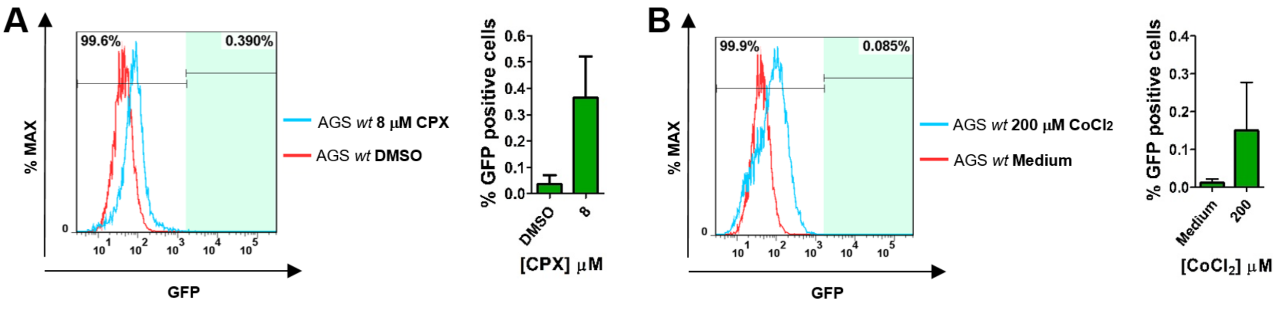

Figure S1: Evaluation of the percentage of GFP positive cells by flow cytometry. Flow cytometry analysis of GFP expression in AGS wt cells **(A)** after 48 h of treatment with 8 μM CPX and **(B)** after 48 h of treatment with 200 μM $CoCl_2$. wt corresponds to the parental cell line (AGS). DMSO or medium were used as a negative control. Results are mean ± SD of at least three independent experiments.

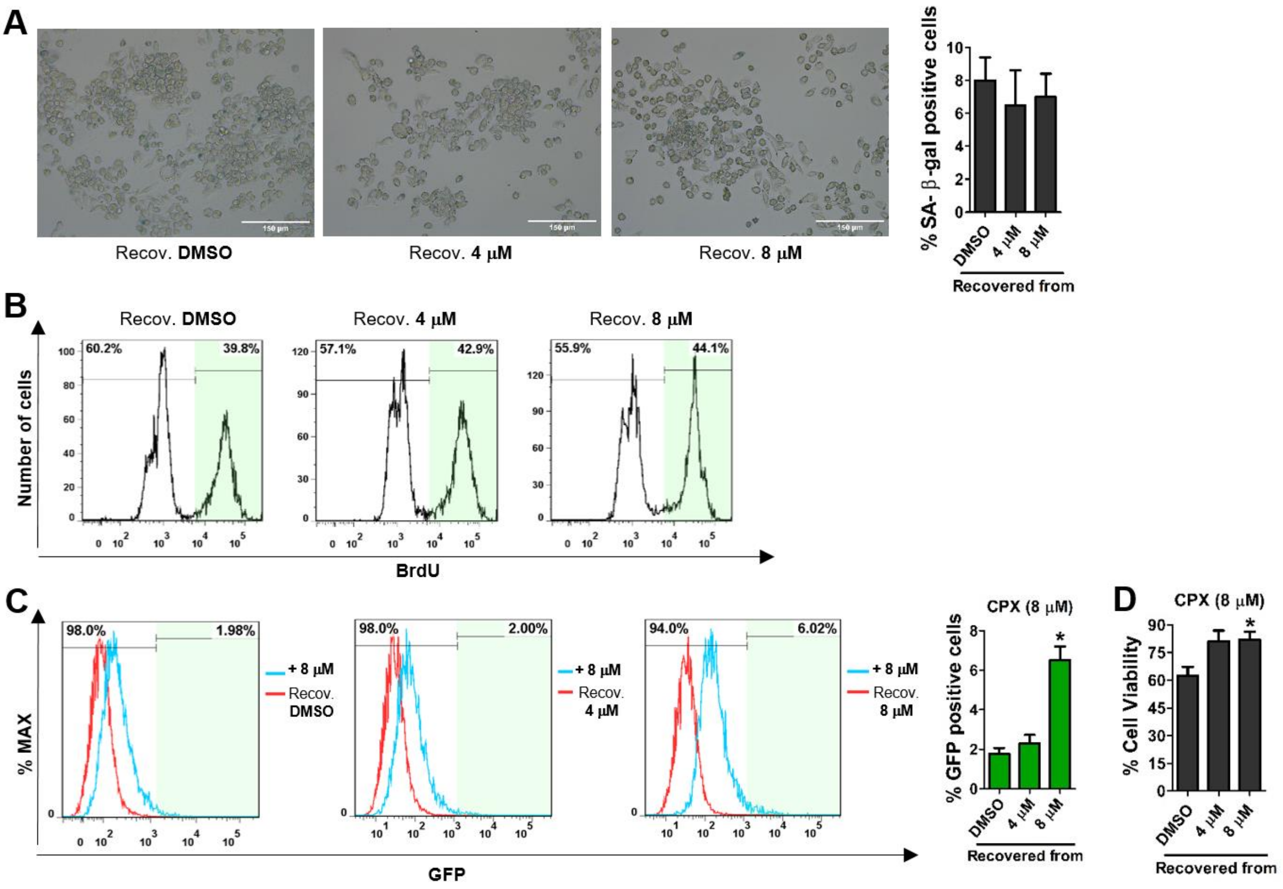

Figure S2: CPX-induced cellular senescence is reversible and cells regain the proliferative ability. **(A)** Percentage of SA-β-gal positive cells in Kato III SORE6− cells 4 weeks after recovery from treatment with DMSO, 4 or 8 μM CPX for 48 h, following representative images of SA-β-gal marking. Scale bar = 150 μm. **(B)** Representative flow cytometry analysis of the percentage of BrdU positive cells in AGS SORE6− cells 4 weeks after recovery from treatment with DMSO, 4 or 8 μM CPX for 48 h. **(C)** Percentage of cell viability after 48 h of treatment of AGS SORE6− and Kato III SORE6− recovered cells with 4.5 μg/mL (IC50) of 5-FU. **(D)** Percentage of GFP-positive cells, following the respective flow cytometry analysis, and (E) cell viability in Kato III SORE6− recovered cells after new treatment with 8 μM CPX for 48 h. DMSO was used as a negative control. Results are mean ± SD of at least three independent experiments. Significant differences * $p \leq 0.05$.

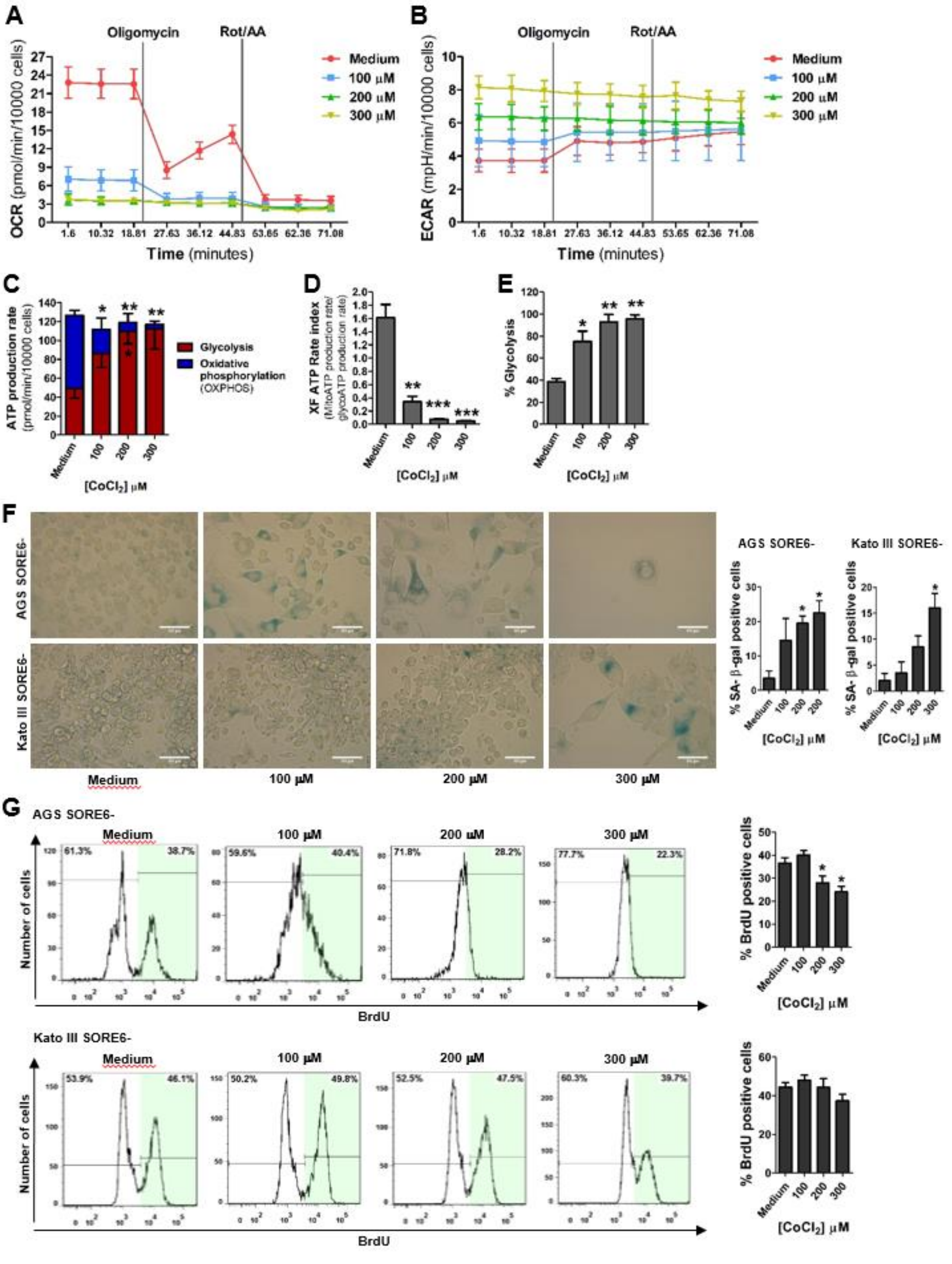

Figure S3: $CoCl_2$ treatment inhibits ATP production by OXPHOS and induces cellular senescence with consequent loss of proliferative activity. **(A)** Representative real-time measurements of the kinetic profile of the OCR, **(B)** representative real-time measurements of the kinetic profile of the ECAR, **(C)** metabolic flux analysis showing quantification of mitochondrial ATP production and glycolytic ATP production, **(D)** ATP production rate index calculated from the data showed in H (i.e., mitochondrial ATP production rate/glycolytic ATP production rate) and **(E)** percentage of gycolysis following treatment with 100, 200 or 300 µM $CoCl_2$ for 48 h. **(F)** Percentage of SA-β-gal positive cells in AGS SORE6− and Kato III SORE6− cells treated with 100, 200 or 300 µM $CoCl_2$ for 48 h, followed by representative images of SA-β-gal marking. Scale bar = 150 µm. **(G)** Percentage of

BrdU positive cells in AGS SORE6− and Kato III SORE6− cells treated with 100, 200 or 300 μM $CoCl_2$ for 48 h, followed by representative flow cytometry analysis. Medium was used as a negative control. Results are mean ± SD of at least three independent experiments. * p ≤ 0.05; ** p ≤ 0.01; *** p ≤ 0.001.

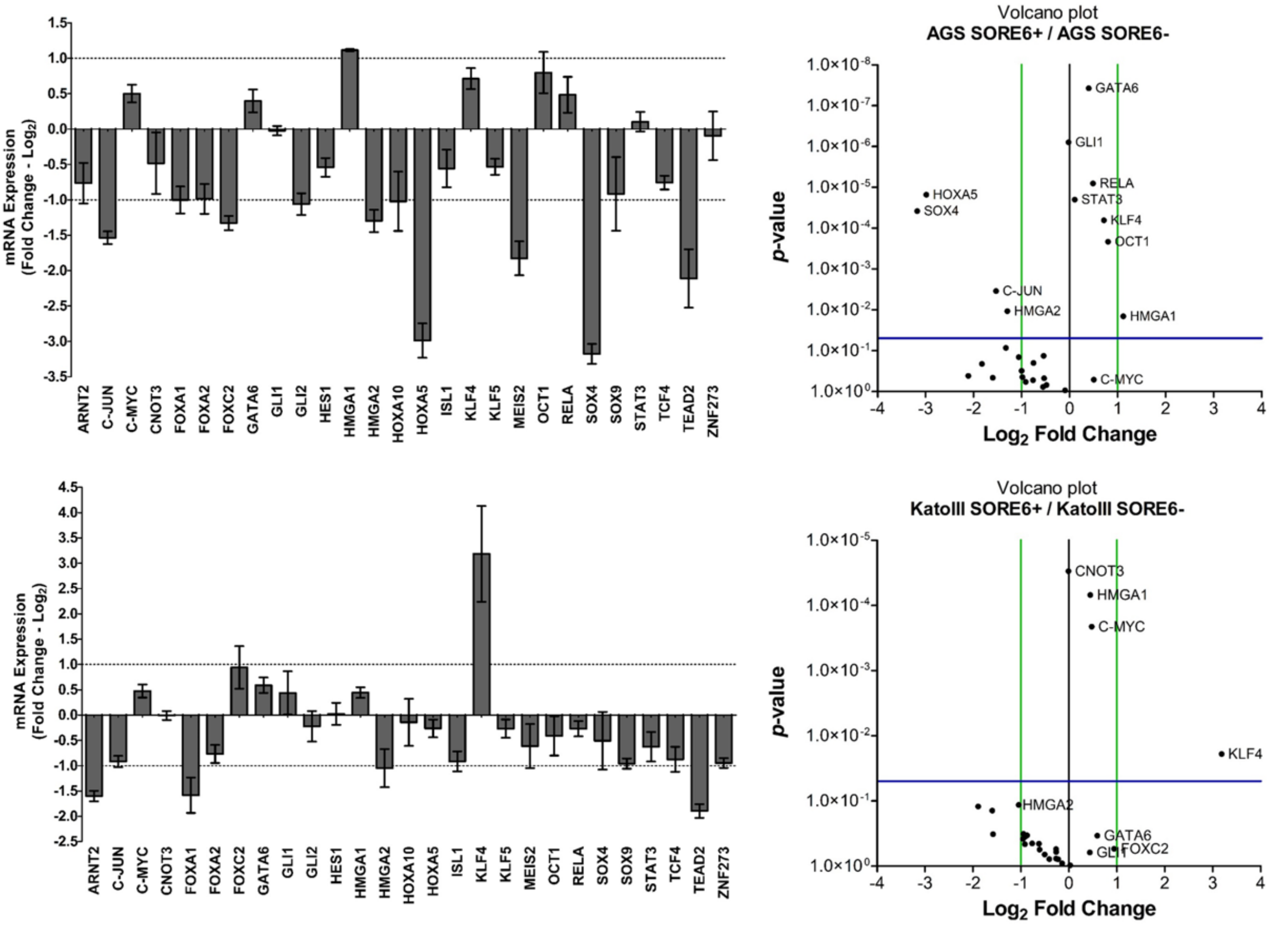

Figure S4: Comparison of the expression of relevant transcription factors between SORE6+ and SORE6− cells. Real-time PCR analysis of mRNA levels of the transcription factors ARNT2, C-JUN, C-MYC, CNOT3, FOXA1, FOXA2, FOXC2, GATA6, GLI1, GLI2, HES1, HMGA1, HMGA2, HOXA10, HOXA5, ISL1, KLF4, KLF5, MEIS2, OCT1, RELA, SOX4, SOX9, STAT3, TCF4, TEAD2 and ZNF273 in AGS SORE6+ and Kato III SORE6+ cells in comparison to AGS SORE6− and Kato III SORE6− cells. Followed by the volcano plot (SORE6+ compared to SORE6− cells) where the horizontal blue line represents the threshold of statistical significance (p = 0.05) and the green lines corresponds to the fold change cut-off ≥ 2. Results were normalized to 18S expression. Results are mean ± SD of at least three independent experiments.

UNCROPPED WESTERN BLOT FIGURES

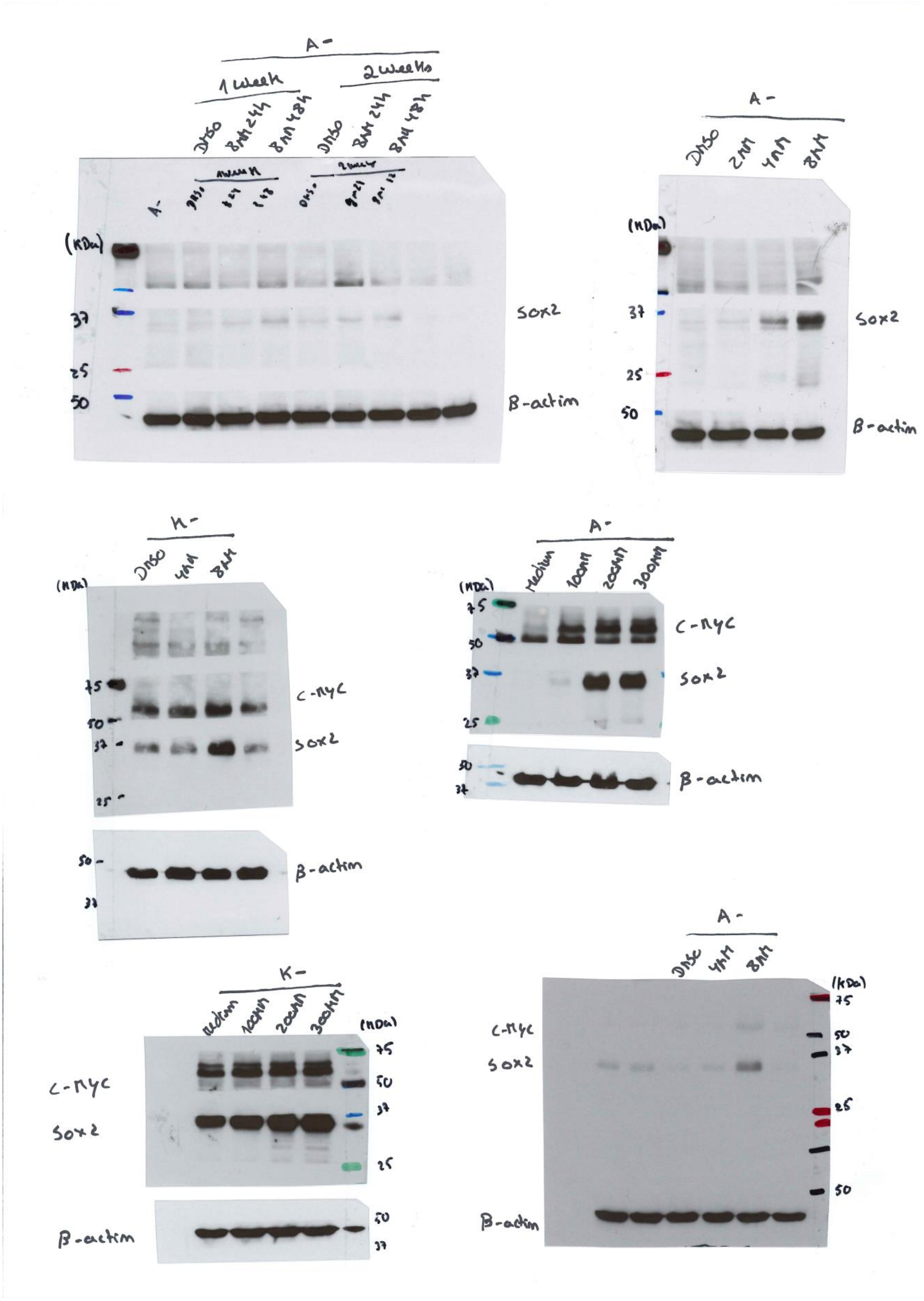
A-
1 Week
2 Weeks
DMSO
(kDa)
37
25
50
Sox2
β-actin
A-
DMSO
(kDa)
37
25
50
Sox2
β-actin
K-
DMSO
(kDa)
75
50
37
25
c-Myc
Sox2
50
37
β-actin
A-
Medium
100nM
200nM
300nM
(kDa)
75
50
37
25
c-Myc
Sox2
50
37
β-actin
K-
Medium
100nM
200nM
300nM
(kDa)
75
50
37
25
c-Myc
Sox2
50
37
β-actin
A-
DMSO
(kDa)
75
50
37
25
50
c-Myc
Sox2
β-actin
